# Machine Learning Prediction of Autism Spectrum Disorder From a Minimal Set of Medical and Background Information

**DOI:** 10.1001/jamanetworkopen.2024.29229

**Published:** 2024-08-19

**Authors:** Shyam Sundar Rajagopalan, Yali Zhang, Ashraf Yahia, Kristiina Tammimies

**Affiliations:** 1 Center of Neurodevelopmental Disorders, Centre for Psychiatry Research, Department of Women’s and Children’s Health, Karolinska Institutet, Solna, Sweden; 2 Department of Highly Specialized Pediatric Orthopedics and Medicine, Astrid Lindgren Children’s Hospital, Karolinska University Hospital, Region Stockholm, Stockholm, Sweden; 3 Institute of Bioinformatics and Applied Biotechnology, Bengaluru, India

## Abstract

**Importance:**

Early identification of the likelihood of autism spectrum disorder (ASD) using minimal information is crucial for early diagnosis and intervention, which can affect developmental outcomes.

**Objective:**

To develop and validate a machine learning (ML) model for predicting ASD using a minimal set of features from background and medical information and to evaluate the predictors and the utility of the ML model.

**Design, Setting, and Participants:**

For this diagnostic study, a retrospective analysis of the Simons Foundation Powering Autism Research for Knowledge (SPARK) database, version 8 (released June 6, 2022), was conducted, including data from 30 660 participants after adjustments for missing values and class imbalances (15 330 with ASD and 15 330 without ASD). The SPARK database contains participants recruited from 31 university-affiliated research clinicals and online in 26 states in the US. All individuals with a professional ASD diagnosis and their families were eligible to participate. The model performance was validated on independent datasets from SPARK, version 10 (released July 21, 2023), and the Simons Simplex Collection (SSC), consisting of 14 790 participants, followed by phenotypic associations.

**Exposures:**

Twenty-eight basic medical screening and background history items present before 24 months of age.

**Main Outcomes and Measures:**

Generalizable ML prediction models were developed for detecting ASD using 4 algorithms (logistic regression, decision tree, random forest, and eXtreme Gradient Boosting [XGBoost]). Performance metrics included accuracy, area under the receiver operating characteristics curve (AUROC), sensitivity, specificity, positive predictive value (PPV), and F1 score, offering a comprehensive assessment of the predictive accuracy of the model. Explainable AI methods were applied to determine the effect of individual features in predicting ASD as secondary outcomes, enhancing the interpretability of the best-performing model. The secondary outcome analyses were further complemented by examining differences in various phenotypic measures using nonparametric statistical methods, providing insights into the ability of the model to differentiate between different presentations of ASD.

**Results:**

The study included 19 477 (63.5%) male and 11 183 (36.5%) female participants (mean [SD] age, 106 [62] months). The mean (SD) age was 113 (68) months for the ASD group and 100 (55) months for the non-ASD group. The XGBoost (termed AutMedAI) model demonstrated strong performance with an AUROC score of 0.895, sensitivity of 0.805, specificity of 0.829, and PPV of 0.897. Developmental milestones and eating behavior were the most important predictors. Validation on independent cohorts showed an AUROC of 0.790, indicating good generalizability.

**Conclusions and Relevance:**

In this diagnostic study of ML prediction of ASD, robust model performance was observed to identify autistic individuals with more symptoms and lower cognitive levels. The robustness and ML model generalizability results are promising for further validation and use in clinical and population settings.

## Key Points

**Question:**

Can a machine learning (ML) model based on minimal background and medical information accurately predict autism spectrum disorder (ASD)?

**Findings:**

This diagnostic study of 30 660 participants using ML prediction of ASD with only 28 features found high predictive accuracy, sensitivity, and specificity. Validation on independent cohorts showed good generalizability, and developmental milestones and eating behavior emerged as important predictive factors.

**Meaning:**

The model developed in this study shows promise in the early identification of individuals with an elevated likelihood of ASD, using minimal information, which could affect early diagnosis and intervention strategies.

## Teaser

This diagnostic study investigates whether machine learning models incorporating easily obtainable measures from medical records and background history data can reliably detect autism spectrum disorder in children.

## Introduction

Autism spectrum disorder (ASD) is a neurodevelopmental condition with challenges in communication and social interaction and the presence of restricted and repetitive behaviors.^1,2^ The estimated prevalence of ASD is approximately 1.0%, but higher rates have been reported, such as 2.78% in a 2023 report from the US.^3^ The current median age of diagnosis is 60.48 (range, 30.90-234.57) months.^4^ Early detection is crucial for targeted early intervention and improved outcomes.^5^

Widely used ASD screening tools such as the Modified Checklist for Autism in Toddlers, Revised With Follow-Up (M-CHAT-R/F),^6^ the Social Attention and Communication Surveillance–Revised,^7^ the Parents’ Evaluation of Developmental Status,^8^ and the Childhood Autism Rating Scale^9^ have been instrumental in identifying individuals with elevated likelihood of ASD.^10^ However, these tools face some challenges related to biases and subjectivity in their measures, which could be related to the interpretation of the questions, cultural differences, and the child’s behavior at the time of assessment.^11,12^ Also, the ASD heterogeneity and associated co-occurring conditions pose further challenges for screening and early diagnosis. The delay in diagnosis can result in a lack of timely interventions, posing a significant family and societal burden.^13^ To mitigate such challenges, new approaches using machine learning (ML) and behavior phenotypes captured using mobile applications^14^ or ML models using electronic health records (EHR)^15^ are emerging but still only used in research settings.

Machine learning methods can potentially develop robust prediction models for ASD using different data modalities.^11,13^ If designed correctly, the power of prediction models comes from their generalization ability to predict unseen individual outcomes reliably. Machine learning methods offer the potential to handle large datasets and identify hidden patterns in the data, quantify behavior phenotypes, and develop automated solutions for scaling.

For ASD, there have been recent advances in using ML methods to predict the condition. For instance, in a recent Israeli study,^16^ routine developmental surveillance data from 1.2 million children were used to develop an automatic prediction tool for ASD diagnosis, which was further compared with M-CHAT screening with superior results. Furthermore, Engelhard et al^15^ proposed an early ASD prediction model from the EHR data collected before the first birthday and showed its promise for integration with other screening tools. Additionally, Onishchenko et al^17^ developed digital biomarkers for ASD from past medical conditions termed the autism comorbid risk score.

We hypothesized that ML models incorporating easily obtainable measures from medical records and background history data can reliably detect ASD. To investigate this hypothesis, we used a large database with multilayered information collected within the Simons Foundation Powering Autism Research for Knowledge (SPARK) study^18^ to develop and test an ML model for early ASD screening. Furthermore, we provided explanatory information on the classification of individuals and investigated differences in several phenotype measures between those participants who were correctly and incorrectly classified using our model.

## Methods

The prediction model development and validation for this diagnostic study followed the Transparent Reporting of a Multivariable Prediction Model for Individual Prognosis or Diagnosis (TRIPOD) reporting guidelines. Ethical approval for the data collection and informed consent were obtained from the participants within the SPARK and Simons Simplex Collection (SSC) projects. The Swedish Ethical Committee approved this study and data analysis in Sweden.

### Study Cohorts

We used 2 available datasets for ASD research, the SPARK^18^ and the SSC^19^ (eFigure 1 in Supplement 1). Both databases have a collection of medical and behavioral data from individuals with ASD and family members living in the US. The SPARK project is coordinated by the Simons Foundation Autism Research Initiative, which collaborates with 31 university-affiliated research clinics in 26 states in the US and has online recruitment. The project was launched in December 2015, and the recruitment of participants is ongoing. All individuals with a professional ASD diagnosis and their families can participate in the study. The clinical validation of the ASD diagnosis within SPARK has shown to be high.^20^

We used the SPARK collection, version 8 (released June 6, 2022), as the primary cohort (Table 1). Version 8 has the following phenotype measures: (1) the basic medical screening measures administered to all individuals with ASD, parents without ASD, and siblings, and (2) the background history responses (eTable 1 in Supplement 1). We furthermore used the reported data on participants’ race and ethnicity to investigate the model performance across these different groups.

**Table 1.** SPARK, Version 8, Study Cohort Demographic Details^a^

Characteristic	Participant group, No. (%)	
	ASD (n = 15 330)	Non-ASD (n = 15 330)
Age, mean (SD), mo	113 (68)	100 (55)
Sex		
Males	11 967 (78.1)	7510 (49.0)
Females	3363 (21.9)	7820 (51.0)
Ethnicity		
Hispanic or Latino	2733 (17.8)	995 (6.5)
Non-Hispanic or Latino	12 597 (82.2)	14 335 (93.5)
Race^b^		
Asian	752 (4.9)	307 (2.0)
American Indian or Alaska Native	492 (3.2)	160 (1.0)
Black or African American	1490 (9.7)	518 (3.4)
Native Hawaiian or Other Pacific Islander	130 (0.8)	46 (0.3)
White	13 342 (87.0)	4954 (32.3)

Abbreviations: ASD, autism spectrum disorder; SPARK, Simons Foundation Powering Autism Research for Knowledge.

^a^ Unless otherwise indicated, data are expressed as number (percentage) of participants. Version 8 was released June 6, 2022.

^b^ Race was not available for all the participants, and multiple race was possible to select in the response.

We included new SPARK participants in the database for ML model testing in version 10 (released July 21, 2023) (eFigure 1 in Supplement 1). Based on the availability of the needed data, we included 11 936 participants (10 476 with ASD and 1460 without ASD) from the 31 384 in the version 10 release not included in the version 8 release.

In addition to the features used for the ML model, we used the following measures available in the SPARK database: the Child Behavior Checklist (CBCL)^21^ for 1 to 5 and 6 to 18 years of age with the total problems *t* score, Full-scale Intelligence Quotient (FSIQ), and Social Communication Questionnaire (SCQ) score.^22,23^ Furthermore, we analyzed the co-occurring diagnosis within behavioral and developmental categories.

The SSC cohort, which consists of 2643 simplex families with 10 474 individuals,^19^ was also used for model testing. The phenotype measures of interest to this study were available for 2854 individuals with ASD. We mapped the predictor variables between the SSC and SPARK cohort databases (eTable 1 in Supplement 1). In total, 14 790 individuals were used in the validation experiments from these 2 cohorts.

We recorded participants’ race and ethnicity. Categories for ethnicity and included Hispanic or Latino and non-Hispanic or Latino; categories for race included Asian, American Indian or Alaska Native, Black or African American, Native Hawaiian or Other Pacific Islander, and White.

### Data Preprocessing and Selection of Features and Participants

We developed early screening prediction models using the measures that can be obtained without elaborative behavior assessments and medical tests before 24 months of age. This inclusion criterion is applied to identify a subset of measures from the basic medical screening and background history administered within the SPARK collection. The selection was based on identifying easily obtainable, noninvasive, parent-reported information in the medical and background questionnaires. The selection of measures used a consensus-based approach prior to the development of the ML model. Twenty-eight variables were selected, of which 11 were present in the basic medical screening and 17 in the background history data (eTable 1 in Supplement 1). Further preprocessing of the features and data is described in the eMethods in Supplement 1. After preprocessing and downsampling for an equal number of individuals with and without ASD, the final dataset size used for model development was 30 660 samples with an equal distribution of participants with and without ASD (eFigure 1 in Supplement 1). Furthermore, age, race, and ethnicity groups were used to investigate ML model performance in secondary analyses.

### Model Development and Validation

We used 4 algorithms—logistic regression, decision tree, random forest, and eXtreme Gradient Boosting (XGBoost)—to train models combining the selected features from the SPARK version 8 cohort. These ML models were developed using the Python scikit-learn library (Python Software Foundation).^24^ The SPARK version 8 cohort was divided into 60% training, 20% validation, and 20% test sets in all experiment conditions.

The validation set facilitated the tuning hyperparameters and was later integrated with the training set for the final model training. The remaining 20% of the data, constituting the unseen test set, was used for model evaluation. Model training was conducted using 10-fold cross-validation.

To avoid overfitting, the XGBoost model was specifically trained using the validation set by using early stopping criteria. Additionally, model parameters were tuned using the bayesian optimization approach within the hyperparameter optimization library^25^ for each fold. The details of all tuned model hyperparameters are provided in the eMethods in Supplement 1.

We implemented the DeLong algorithm^26^ to compute the area under the receiver operating characteristics curve (AUROC) with a 95% CI for model evaluation. To assess the effect of each predictor on the model performance, we computed the mean Shapley additive explanations (SHAP) values.^27^ Furthermore, various other performance metrics were calculated, including accuracy, AUROC, sensitivity, specificity, positive predictive value (PPV), and F1 score. We report the mean values for these measures across 10-fold validation. The best-performing model was chosen for further testing using other datasets, and performance metrics on validation were calculated for the first fold only. Furthermore, we have computed calibrated precision (PPV) and F1 scores to study the model performance in scenarios of class imbalance.^28^

Additional details about the model development, further experiments, including sex- and age-specific models, and evaluation are provided in eMethods in Supplement 1.

### SHAP Computation 

We applied the SHAP^27^ explainable approach to study the influence of predictors toward ASD classification using the python SHAP module’s TreeExplainer for the XGBoost model generated for the first fold. For categorical features, we aggregated the SHAP values of their corresponding one hot encoded features to determine the overall SHAP value. For numerical features, the SHAP values are directly obtained.

### Statistical Analysis

All statistical analyses were performed in R, version 4.2.2 (R Project for Statistical Computing). We examined clinical measures between individuals correctly predicted with ASD or without ASD and those incorrectly predicted in the SPARK, version 10 sample using quantitative measures: CBCL *t* scores, FSIQ, and SCQ scores. We assessed statistical significance using 2-sided Wilcoxon rank sum tests with the stats package in R, due to the nonnormal distribution of the data, as confirmed by the Shapiro-Wilk test. Additionally, we investigated whether other diagnoses were more prevalent in participants identified as having ASD compared with those identified as not having ASD using χ^2^ tests with Bonferroni-adjusted *P* values for significance testing, and odds ratios with 95% CIs using the epitools package, version 0.5-10.1. We visualized the forest plot using forestploter package, version 1.1.2. Two-sided *P* < .05 indicated statistical significance.

## Results

### ASD Prediction Model

**Figure 1.**
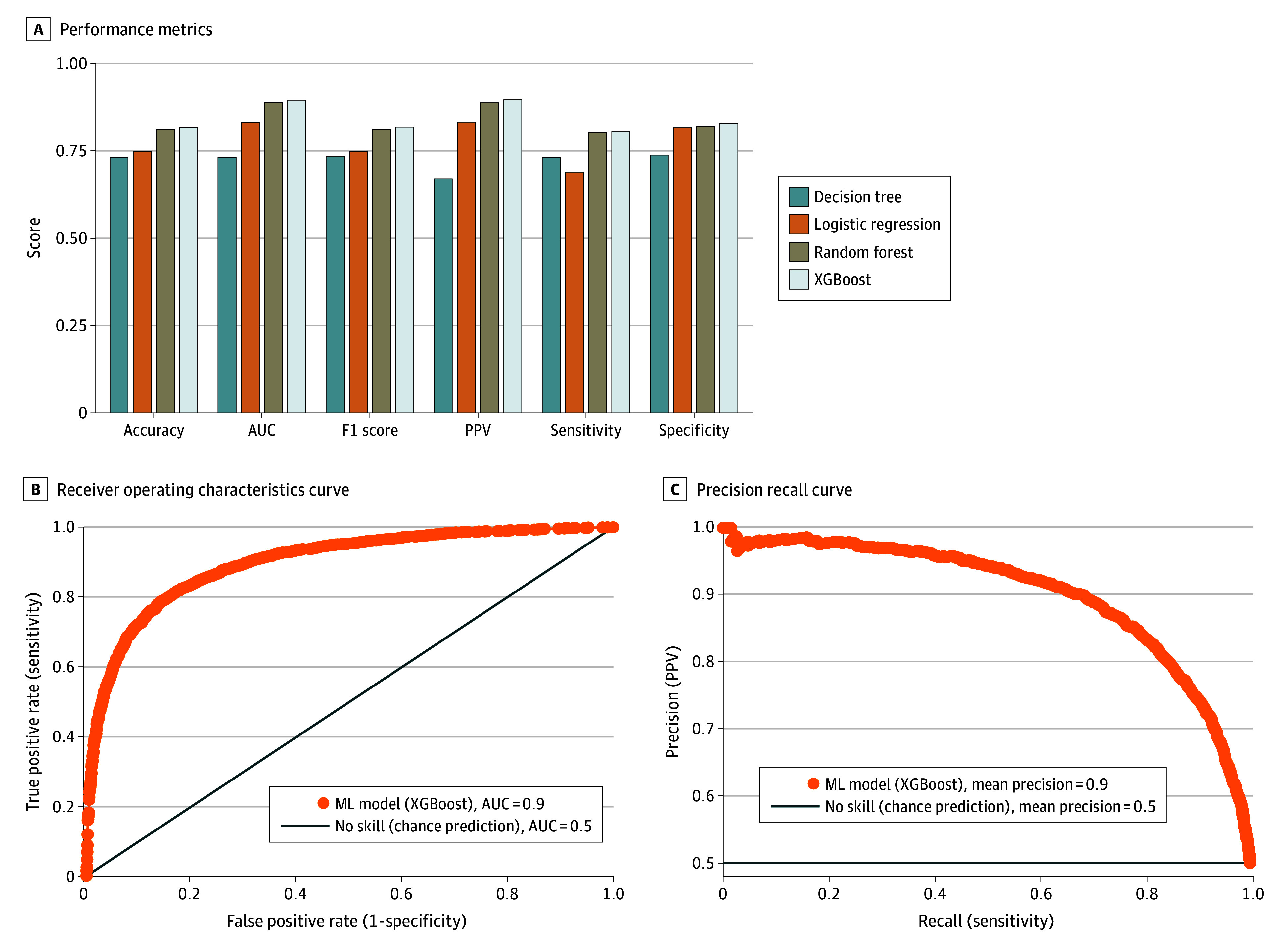
The Performance of Machine Learning (ML) Algorithms Using the Combined Medical Screening and Background History Measures A, Performance metrics for each of the 4 ML models—decision tree, logistic regression, random forest, and eXtreme Gradient Boosting (XGBoost)—developed. B, The receiver operating characteristic curve for the best-performing XGBoost algorithm. C, The precision-recall curve for the best-performing XGBoost algorithm. AUR indicates area under the curve; PPV, positive predictive value.

The study included 30 660 participants for the ML model development, including 15 330 participants in ASD and non-ASD groups. The study cohort included 19 477 (63.5%) male and 11 183 (36.5%) female participants (mean [SD] age, 106 [62] months) (Table 1). In terms of ethnicity, 3728 (12.2%) were Hispanic or Latino and 26 932 (87.8%) were non-Hispanic or Latino. In terms of race, 652 (2.1%), American Indian or Alaska Native; 2008 (6.5%), Black or African American; 176 (0.6%), Native Hawaiian or Other Pacific Islander; and 18 296 (59.7%), White. The mean (SD) age for the ASD group was 113 (68) months; for the non-ASD group, 100 (55) months. In this sample of 30 660 SPARK participants with both medical screening and background history measures available, we trained 4 different ML models using 28 selected features (eFigure 1 and eTable 1 in Supplement 1). All the trained models showed good performance metrics (Table 2 and Figure 1A). However, the XGBoost algorithm achieved the best performance with a mean AUROC of 0.895 (σ = 0.004 [σ value indicates the SD]) in the test set across 10-fold validations. The receiver operating characteristics curve and the precision recall curves for the best-performing XGBoost algorithm are shown in Figure 1, B and C, respectively. The XGBoost-based model was chosen for the rest of the analysis and termed AutMedAI.

**Table 2.** Performance of ML Algorithms on a Combined Dataset of Medical Screening and Background History Information

ML Algorithm	Accuracy	AUROC^a^	Sensitivity	Specificity	Positive predictive value	F1 Score^b^
Logistic regression	0.752	0.831 (σ = 0.004)	0.689	0.816	0.832	0.735
Decision tree	0.732	0.732 (σ = 0.006)	0.729	0.735	0.670	0.731
Random forest	0.812	0.889 (σ = 0.004)	0.803	0.820	0.888	0.810
XGBoost^c^	0.817	0.895 (σ = 0.004)	0.805	0.829	0.897	0.815
AutMedAI validation experiment^d^						
SPARK version 10 cohort	0.789	0.790	0.789	0.791	0.964	0.868
AutMedAI validation experiment calibrated^d^						
SPARK version 10 cohort	0.789	0.790	0.789	0.791	0.729	0.790

Abbreviations: AUROC, area under the receiver operating characteristics curve; ML, machine learning; SPARK, Simons Foundation Powering Autism Research for Knowledge.

^a^ Parenthetical value (σ) value indicates the SD of AUROC across 10-fold cross-validation.

^b^ F1 scores range from 0 to 1, with higher scores indicating better balance between precision and recall, thus representing more accurate and reliable classification performance.

^c^ Balanced dataset, pi_0 = 0.5.

^d^ The XGBoost-based model was chosen for the rest of the analysis and termed AutMedAI.

Additional experiments conducted using combined medical screening and background history without the sex variable achieved an AUROC of 0.885 (σ = 0.004) (eTable 2 in Supplement 1). Also, the AUROC scores stratified across age groups of individuals at the time of evaluation were 0.868 for 0 to 2 years, 0.920 for 2 to 4 years, and 0.906 for 4 to 10 years (eTable 3 in Supplement 1). The most important predictors for each age group varied and can be found in the eFigures 2 to 4 in Supplement 1.

Our additional model development exercises using only the medical screening items resulted in inferior model performance, with the highest AUROC being 0.783 (σ = 0.005) (eTable 2 in Supplement 1). However, the background history items performed similarly to the combined model, with the highest AUROC of 0.870 (σ = 0.004) (eTable 2 in Supplement 1).

### Prediction Model Testing

**Figure 2.**
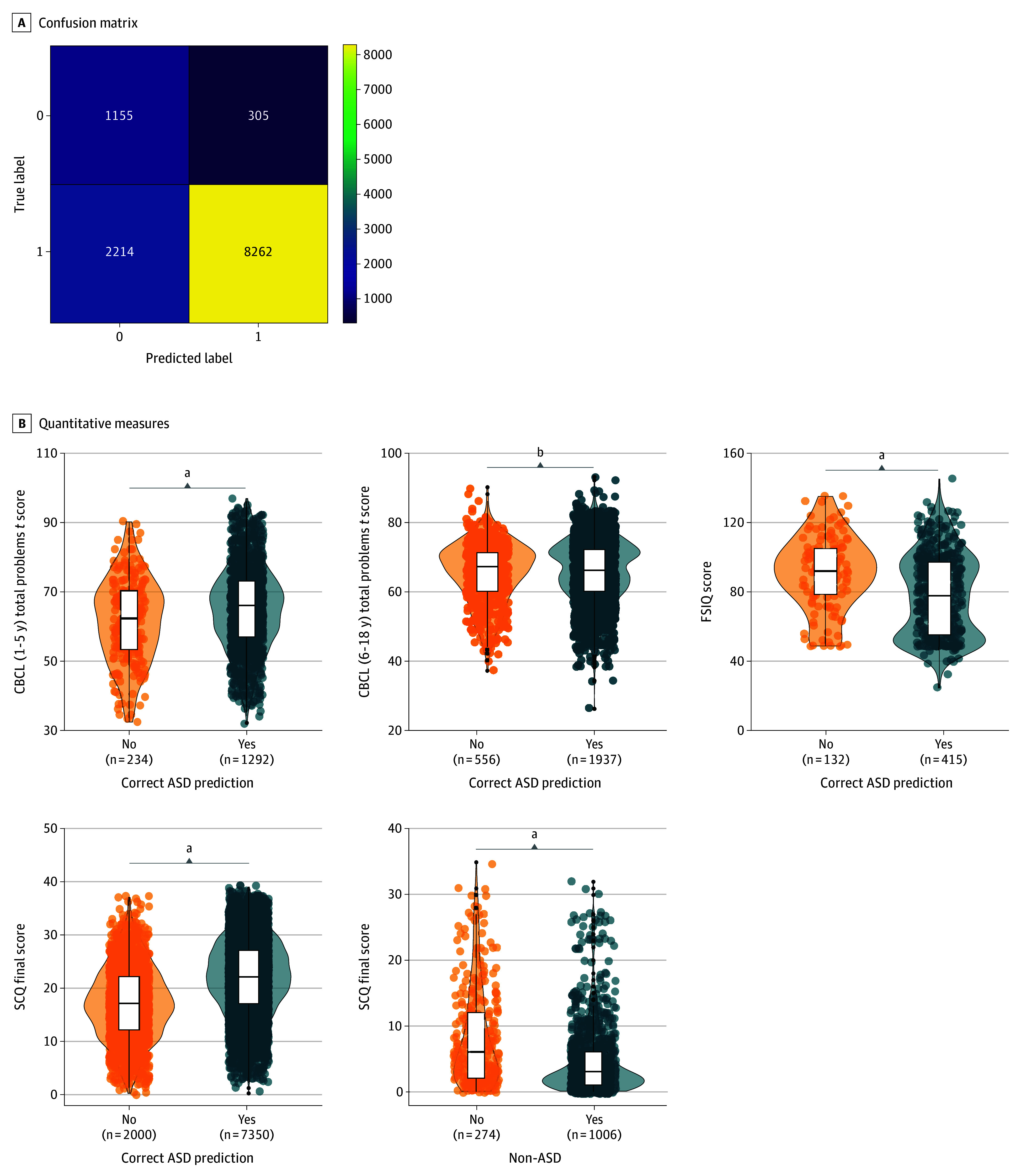
The Model Validation and Phenotype Associations Using the Simons Foundation Powering Autism Research for Knowledge Version 10 Cohort A, The confusion matrix shows the model performance by indicating the number of participants correctly identified with or without autism spectrum disorder (ASD) in each group. Label 1 indicates ASD and 0 indicates non-ASD. The numbers in each cell correspond to the number of samples. B, Differences in Child Behavior Checklist (CBCL) *t* score for 1 to 5 and 6 to 18 years of age (range, 50-100, with higher scores indicating more severe behavioral and emotional problems), Full-Scale IQ (FSIQ), and Social Communication Questionnaire (SCQ; range, 0-39, with higher scores indicating more social communication challenges) among those predicted to have ASD with the model separated by correct and incorrect predictions. ^a^*P* < .001. ^b^Not significant.

To ensure model generalizability, we tested the model performance on 11 936 participants from SPARK version 10 (only new recruits) and 2854 participants with ASD from SSC (eFigure 1 in Supplement 1), in total 14 790 participants. Our main testing cohort was the SPARK version 10. When testing the model for 11 936 participants, including 10 476 in the ASD group and 1460 in the non-ASD group, we correctly identified 9417 participants with or without ASD (78.9%). Among the children with ASD, the model correctly identified 8262 (78.9%) individuals (Figure 2A). The AUROC score of the validation was 0.790. The results of other metrics are shown in Table 2. The AUROC score for the SPARK version 10 cohort validation without the sex variable was 0.781 (eTable 4 in Supplement 1). The AUROC scores for different age groups were 0.807 (0-2 years), 0.798 (2-4 years), and 0.791 (4-10 years) (eTable 4 in Supplement 1). The validation scores for other evaluation metrics stratified by race and sex were robust and are shown in eTable 4 in Supplement 1.

Next, we analyzed SHAP values for each participant within the SPARK version 10 cohort to determine the features most influential in ASD prediction (eFigure 5 in Supplement 1). Notably, features like problems with eating foods, age at first use of short phrases or sentences including an action word, age at first construction of longer sentences, age at achieving bowel training, and age at first smile emerge as the most significant predictors, as evidenced by their high SHAP values.

The influence of the top 20 features toward correct ASD prediction for 3 children with ASD correctly predicted by the model (eFigure 6 in Supplement 1) and for 3 children with ASD incorrectly predicted by the model are shown in eFigure 6 in Supplement 1. A decision plot of all the features across individuals is shown in eFigure 7 in Supplement 1.

Furthermore, we tested the model in 2854 individuals with ASD from the SSC cohort. Our experiment resulted in a sensitivity score of 0.680. The results of other metrics are shown in eTable 5 in Supplement 1. Since the SSC dataset in this study contained only children with ASD, the AUROC, specificity, and F1 scores were not calculated.

### Phenotype Associations With Predicted Labels

We studied the association between model predictions (correctly or incorrectly as ASD or non-ASD) and clinical measures to understand the usefulness of our model. We investigated CBCL scores, FSIQ, and SCQ scores of individuals belonging to ASD and non-ASD groups predicted by the model (eTable 6 in Supplement 1 and Figure 2B). The information about the cohort sample size, missing data, and *P* values are shown in eTable 6 in Supplement 1. The correctly predicted ASD group had significantly more problems at 1 to 5 years of age as indicated by the CBCL *t* scores (2-sided Wilcoxon rank sum test, *P* < .001), significantly lower FSIQ (2-sided Wilcoxon rank sum test, *P* < .001), and more social communication difficulties as indicated by the SCQ (2-sided Wilcoxon rank sum test, *P* < .001) as the incorrectly predicted (as non-ASD) ASD group. However, we did not find a significant difference between the 2 groups on CBCL *t* scores at 6 to 18 years of age (2-sided Wilcoxon rank sum test, *P* = .73).

**Figure 3.**
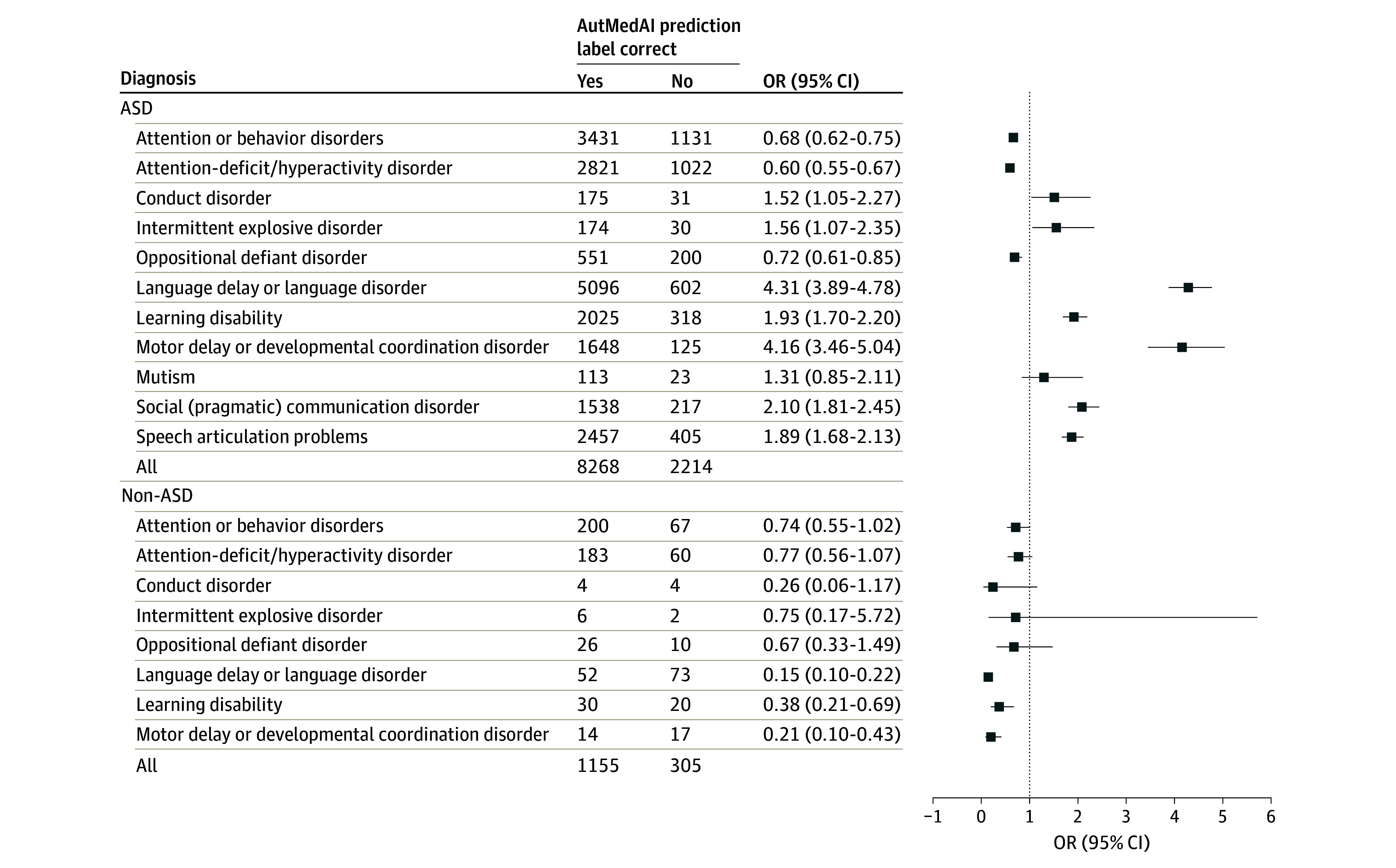
Prevalence of the Other Behavioral and Developmental Diagnoses Data are given among individuals correctly vs incorrectly predicted to have autism spectrum disorder (ASD) and correctly vs incorrectly predicted to have no ASD. OR indicates odds ratio.

When we tested the prevalence of the other behavioral and developmental diagnoses among the groups, we found that the group with correctly predicted ASD had a lower rate of diagnosis of attention-deficit/hyperactivity disorder (Figure 3 and eTable 7 in Supplement 1). As expected, based on the predictors, all developmental diagnoses were more common in the group with correctly labeled ASD than in the group with incorrect predictions (Figure 3 and eTable 7 in Supplement 1).

Furthermore, we compared the same measures within participants without ASD, of whom 1155 were predicted correctly (as non-ASD), and 305 were predicted incorrectly (as ASD). From the quantitative clinical measures, only SCQ score was available for this group. We demonstrate that the incorrectly labeled individuals in the non-ASD group (predicted as having ASD) had a significantly higher SCQ score (*P* < .001) than those correctly labeled as not having ASD (Figure 2B). Similarly, incorrectly labeled individuals (as having ASD) in the non-ASD group were more likely to have other developmental diagnoses (Figure 3 and eTable 7 in Supplement 1). These results indicate that the model identified ASD in individuals with more severe symptoms and more general developmental issues. The model also revealed a group of participants without ASD but with higher ASD traits measured by the SCQ score.

## Discussion

In this diagnostic study, we used the currently largest database for ASD research,^18^ SPARK, to develop a prediction model, AutMedAI, to screen for ASD in infancy and early childhood using minimal background, developmental, and medical information. We found a robust performance of the model with an AUROC of 0.895 and correctly identifying 78.9% of newly introduced participants as either having ASD or not having ASD. Among the approximately 21% of participants who were incorrectly identified with or without ASD, significant differences were found when compared with those correctly identified, showcasing that the model can identify those individuals with more symptoms, especially in communication skills and social functioning, as measured by the screening SCQ.

Numerous tools exist for ASD screening and prediction, ranging from questionnaires to advanced ML-based digital platforms.^2,10^ While many questionnaire-based tools are used in clinical practices, most ML-based digital platforms are currently explored in research settings. The efficacy of these tools often varies based on the age group assessed and differences in race and ethnicity, sex, and the cutoff scores used for performance metrics. For example, M-CHAT-R/F reported an AUROC of 0.907, with a sensitivity of 0.73 and specificity of 0.83, using a cutoff score of 3 for screening toddlers aged 18 to 24 months.^6^ The SCQ score at the cutoff of 15 yields an AUROC of 0.80, sensitivity of 0.69, and specificity of 0.71 when administered to children aged 4 to 12 years.^29^ Similarly, newly emerging digital screening tools using ML models demonstrate acceptable performance for early detection. The digitization enables using data from different modalities, such as EHR for 1 year or younger (sensitivity, 59.8%; specificity, 81.0%; PPV, 17.6%),^15^ behavioral phenotypes captured via mobile devices for 17 to 36 years of age (AUROC, 0.90; sensitivity, 87.8%; specificity, 80.8%; PPV, 40.6%),^14^ and surveillance data to build screening applications for 18 to 24 months of age (AUROC, 0.8; sensitivity, 45.1%; specificity, 95.0%).^16^ Our proposed ML-based model using combined basic medical screening and background history information achieved good performance with an AUROC of 0.895, sensitivity of 0.805, specificity of 0.829, and PPV of 0.897 showing overall strong model performance.

While some of the existing questionnaire-based screen tools are sensitive to age, sex, and race and ethnicity,^10^ the performance of our proposed model is robust in handling this diversity. For example, the difference in the AUROC, sensitivity, specificity, and PPV scores for our best-performing ML model with and without the sex variable is less than 2% for the SPARK version 10 validation cohort. Similarly, the model performance is consistent across different age groups, indicating the model’s robustness for these diverse populations. We did not see any large differences within the different race groups either.

Different modalities used in screening tools, the diversity in population, the administration protocol, and the cutoff scores used to evaluate performance make it difficult to compare tools. Depending on the input data used for screening, different tools may apply to different age groups. Our proposed model, using only basic medical and background information, can be used to screen children at a very early age. Our study found that when the model is used in cohorts of children younger than 2 years, the ML model achieves an AUROC of 0.868. The challenges associated with the heterogeneity of autism, the applicability of different screening instruments in various settings, and the quantification and scaling abilities of digital tools may warrant using a combination of such multimodal screening tools at scale for reliable and robust prediction accuracy.

Our developed model has the potential for clinical use as a noninvasive ASD screening tool. In addition to robust performance, identifying discriminating predictors, such as past medical conditions and phenotype behaviors, for ASD detection is crucial for the clinical adaptation of such screening tools. Explanatory ML models can inform clinicians about the underlying factors leading to ASD detection. Further, they can assist in targeted intervention and follow-up.^30^

### Limitations

We acknowledge several limitations in our approach. Some of the features in our model vary widely within typically developing children, such as the timing of learning to speak and toilet training. Our model needs further validation for its generalizability across different population types in multiple sites. Also, combination with the additional tools using objective measurements such as eye-tracking or brain-based biomarkers should be considered in the future.^31^

## Conclusions

This diagnostic study represents a significant development in applying ML to ASD prediction. We found that early medical information in child care clinics can be used to screen for those with a higher probability of being diagnosed with ASD. The robustness and ML model generalizability results are promising for further validation and use in clinical and population settings.

## Notes

Supplement 1. **eMethods.** Data Preprocessing and Model Training and Development **eTable 1.** List of Predictors From the SPARK Version 8 Cohort Used for Model Development and Validation **eTable 2.** Performance of ML Algorithms **eTable 3.** Performance of the XGBoost Algorithm AutMedAI Stratified by Age **eTable 4.** Performance of the AutMedAI Assessed Using the SPARK Version 10 Cohort **eTable 5.** Performance of AutMedAI Using the SSC Cohort With 2854 Individuals With ASD **eTable 6.** Differences in Quantitative Measures for Those in the ASD Group (Correct vs Wrong Prediction) and Those in the Non-ASD Group (Correct vs Wrong Prediction) **eTable 7.** Frequency and Odds Ratio for Different Behavioral and Developmental Diagnoses Between Those in the ASD Group (Correct vs Wrong Prediction) and Those in the Non-ASD Group (Correct vs Wrong Prediction) **eFigure 1.** Overview of the Study and the ML Model Development **eFigure 2.** Influencing Predictors for Autism Detection in the Group Aged 0 to 2 Years **eFigure 3.** A Beeswarm Plot for All Test Set Samples in the SPARK Version 10 Cohort Aged 2 to 4 Years **eFigure 4.** A Beeswarm Plot for All Test Set Samples in the SPARK Version 10 Cohort Aged 4 to 10 Years **eFigure 5.** A Beeswarm Plot for Test Set Samples of the SPARK Version 10 Cohort **eFigure 6.** The Influence of the Top 20 Features for 6 Individuals in the SPARK Version 10 Cohort **eFigure 7.** Decision Plot Showing the Influence of Features on Model Prediction

Supplement 2.
Data Sharing Statement
