# Supplementary material for: Machine Learning Prediction of Autism Spectrum Disorder From a Minimal Set of Medical and Background Information
Source: JAMA Netw Open. 2024 Aug 19;7(8):e2429229. doi: 10.1001/jamanetworkopen.2024.29229 (PMC11333987; doi:10.1001/jamanetworkopen.2024.29229)
Supplement: Supplement 1. — eMethods. Data Preprocessing and Model Training and Development eTable 1. List of Predictors From the SPARK Version 8 Cohort Used for Model Development and Validation eTable 2. Performance of ML Algorithms eTable 3. Performance of the XGBoost Algorithm AutMedAI Stratified by Age eTable 4. Performance of the AutMedAI Assessed Using the SPARK Version 10 Cohort eTable 5. Performance of AutMedAI Using the SSC Cohort With 2854 Individuals With ASD eTable 6. Differences in Quantitative Measures for Those in the ASD Group (Correct vs Wrong Prediction) and Those in the Non-ASD Group (Correct vs Wrong Prediction) eTable 7. Frequency and Odds Ratio for Different Behavioral and Developmental Diagnoses Between Those in the ASD Group (Correct vs Wrong Prediction) and Those in the Non-ASD Group (Correct vs Wrong Prediction) eFigure 1. Overview of the Study and the ML Model Development eFigure 2. Influencing Predictors for Autism Detection in the Group Aged 0 to 2 Years eFigure 3. A Beeswarm Plot for All Test Set Samples in the SPARK Version 10 Cohort Aged 2 to 4 Years eFigure 4. A Beeswarm Plot for All Test Set Samples in the SPARK Version 10 Cohort Aged 4 to 10 Years eFigure 5. A Beeswarm Plot for Test Set Samples of the SPARK Version 10 Cohort eFigure 6. The Influence of the Top 20 Features for 6 Individuals in the SPARK Version 10 Cohort eFigure 7. Decision Plot Showing the Influence of Features on Model Prediction [file jamanetwopen-e2429229-s001.pdf]

Supplementary Online Content

Rajagopalan SS, Zhang Y, Yahia A, Tammimies K. Machine learning prediction of autism spectrum disorder from a minimal set of medical and background information. *JAMA Netw Open*. 2024;7(8):e2429229. doi:10.1001/jamanetworkopen.2024.29229

eMethods. Data Preprocessing and Model Training and Development
eTable 1. List of Predictors From the SPARK Version 8 Cohort Used for Model Development and Validation
eTable 2. Performance of ML Algorithms
eTable 3. Performance of the XGBoost Algorithm AutMedAI Stratified by Age
eTable 4. Performance of the AutMedAI Assessed Using the SPARK Version 10 Cohort
eTable 5. Performance of AutMedAI Using the SSC Cohort With 2854 Individuals With ASD
eTable 6. Differences in Quantitative Measures for Those in the ASD Group (Correct vs Wrong Prediction) and Those in the Non-ASD Group (Correct vs Wrong Prediction)
eTable 7. Frequency and Odds Ratio for Different Behavioral and Developmental Diagnoses Between Those in the ASD Group (Correct vs Wrong Prediction) and Those in the Non-ASD Group (Correct vs Wrong Prediction)
eFigure 1. Overview of the Study and the ML Model Development
eFigure 2. Influencing Predictors for Autism Detection in the Group Aged 0 to 2 Years
eFigure 3. A Beeswarm Plot for All Test Set Samples in the SPARK Version 10 Cohort Aged 2 to 4 Years
eFigure 4. A Beeswarm Plot for all Test Set Samples in the SPARK Version 10 Cohort Aged 4 to 10 Years
eFigure 5. A Beeswarm Plot for Test Set Samples of the SPARK Version 10 Cohort
eFigure 6. The Influence of the Top 20 Features for 6 Individuals in the SPARK Version 10 Cohort
eFigure 7. Decision Plot Showing the Influence of Features on Model Prediction

This supplementary material has been provided by the authors to give readers additional information about their work.

© 2024 Rajagopalan SS et al. *JAMA Network Open*.

eMethods. Data Preprocessing and Model Training and Development

Data pre-processing

The following data pre-processing and individual selection measures were done. In the medical screening data, the presence of 'null' for a variable indicates the absence of a condition. Therefore, we replaced the 'null' values with 0. The samples with a missing value for any variables were removed from the final set before model training. We converted all categorical predictor variables into one-hot encoding vectors, while numerical values are standardized to zero mean and unit variance (see Table S2).

We utilized data augmentation methods to handle the sample size imbalance between autistic and non-autistic samples but did not find significant performance improvements compared to a down-sampling method. Therefore, we employed a down-sampling strategy by choosing an equal number of non-autistic participants corresponding to the autistic participant sample size at random.

The predictor variable values were standardized between the SPARK and SSC cohorts prior to the model testing. There were two features - "*Age in months when first combined words into short phrases or sentences with an action word*" and "*Is child/dependent a twin or part of a multiple birth?*" – for which there were no equivalent mappings in the SSC database. Therefore, we coded these features as unavailable and used the remaining features for validation. The variable mapping and their value standardization are provided in Table S2.

Model Training Information

Data splits for each fold are done using the same random_state = 1 for reproducibility across different runs of experiments. The scikit-learn ColumnTransformer is used with Standard Scaler and one-hot encoding for standardizing the numerical features and encoding categorical features respectively. Note that the transforms are applied using the ColumnTransformer module with standard_scaler and onehot_encoder. The scaler was fit to the train data only, and the resulting scaler was used to transform the test data. The value for the gest_age measure in the SPARK v8 cohort is available only if preterm birth is selected. Therefore, for those samples that are not preterm, this field is blank. We replaced it with 40 weeks before model training. Bayesian optimization is used for hyperparameter tuning for each fold during the model training. The hyperparameters tuned for different algorithms are given below.

<u>Logistic Regression</u>

C: [0.001, 0.005, 0.01, 0.05, 0.1, 0.5, 1]

<u>Decision Tree</u>

criterion: ['gini', 'entropy', 'log_loss'],
max_depth: [50, None]

<u>Random Forest</u>

n_estimators: [50, 100],
max_depth: [50, None]

XGBoost

learning_rate: [0.0001, 0.001, 0.01, 0.1, 1],
max_depth: range(3, 21, 3)
gamma: [i / 10.0 for i in range(0, 5)],
colsample_bytree: [i / 10.0 for i in range(3, 10)],
reg_alpha: [1e-5, 1e-2, 0.1, 1, 10, 100],
reg_lambda: [1e-5, 1e-2, 0.1, 1, 10, 100], n_estimators: range(0, 100, 10)

Optimal parameters:
Logistic Regression
C: 1

Decision Tree
criterion: 'gini',
max_depth: 50

Random Forest
n_estimators: 100
max_depth: 50

XGBoost
learning_rate: 0.1,
max_depth: 18
gamma: 0.1,
colsample_bytree: 0.3,
reg_alpha: 10,
reg_lambda: 0.1

Additional model development exercises

We conducted two additional model development exercises to test the effectiveness of individual medical screening and background history items. We did similar model training with the same dataset but only using the items from medical screening or background history. Additionally, we conducted a sensitivity analysis for the "sex" predictor variable in our cohort. We did identical model training and evaluation procedures without the "sex" variable using the SPARK v8 cohort and validated its generalizability using the SPARK v10 and SSC cohorts. To study the sensitivity of model performance and to determine discriminating predictors across different age groups at their evaluation, we analyzed the model performance with participants at 0 – 2 years, 2 – 4 years, and 4 – 10 years, race, and sex.

eTable 1. List of Predictors From the SPARK Version 8 Cohort Used for Model Development and Validation

A list of predictors from the SPARK v8 cohort used for model development and validation, and information on these variables is also provided in Simons Simplex Cohort. The mean and the range of values for the sample set used for model development and validation are shown for all numerical variables.

Item No.	Predictor Variable	Description	Category	Mean	SSC Predictor Variable	Data Transformation
**1**	sex	Sex assigned at birth	basic_medical_screening	NA	sex	One-hot encoded
**2**	gest_age	How many weeks (gestational age) was child/dependent when he/she was born?	basic_medical_screening	33.6 wk (20.0 – 36.0)	gestational_age_weeks	z-score normalization
**3**	eating_probs	Problems with eating foods - not diagnosed by a professional	basic_medical_screening	NA	eating_disorder_proband	One-hot encoded
**4**	feeding_dx	Feeding/eating problems	basic_medical_screening	NA	feeding_difficulty	One-hot encoded
**5**	med_cond_birth	Birth or pregnancy complications	basic_medical_screening	NA	first_column_birth_complications or second_column_birth_complications or third_column_birth_complications or fourth_column_birth_complications or fifth_column_birth_complications or sixth_column_birth_complications	One-hot encoded
**6**	birth_oth_calc	Self-reported birth complications not represented in the current coding	basic_medical_screening	NA	coded as 0 to indicate the absence of a condition as defined in SPARK	One-hot encoded
**7**	med_cond_birth_def	Birth defects	basic_medical_screening	NA	abnormal_shape_polydactyly_proband or cleft_lip_palate_proband or congenital_heart_defect_proband or kidney_defect_proband or open_spine_proband	One-hot encoded
**8**	med_cond_growth	Growth conditions (including height, weight, and head size)	basic_medical_screening	NA	physical_anomalies_heart or physical_anomalies_kidney or physical_anomalies_other or physical_anomalies_polydactyly	One-hot encoded
**9**	growth_oth_calc	Growth conditions not represented in the current coding	basic_medical_screening	NA	birth_dev_other	One-hot encoded
**10**	med_cond_neuro	Neurological conditions	basic_medical_screening	NA	cerebral_palsy_proband or congenital_rubella_proband or cranial_nerve_disorder_proband or hydrocephalus_proband or landau_kleffner_syndrome_proband or migraines_proband or neurofibromatosis_proband or	One-hot encoded

					other_neurological_disorder_proband or seizures_proband or tuberous_sclerosis_proband	
**11**	med_cond_visaud	Vision or hearing conditions	basic_medical_screening	NA	hearing or vision	One-hot encoded
**12**	mother_highest_education	Highest level of education of mother/guardian:	background_history	NA	highest_edu_mother	One-hot encoded
**13**	father_highest_education	Highest level of education of father/guardian:	background_history	NA	highest_edu_father	One-hot encoded
**14**	annual_household_income	Annual household income	background_history	NA	annual_household	One-hot encoded
**15**	smiled_age_mos	Age in months when first smiled	background_history	2.9 mo (1 – Not yet)	age_smiled	z-score normalization
**16**	sat_wo_support_age_mos	Age in months when first sat without support	background_history	6.2 mo (1 – Not yet)	age_sat_wo_support	z-score normalization
**17**	crawled_age_mos	Age in months when first crawled	background_history	8.4 mo (1 – Not yet)	age_crawled	z-score normalization
**18**	walked_age_mos	Age in months when first walked alone	background_history	13.2 mo (1 – Not yet)	age_walked_alone	z-score normalization
**19**	fed_self_spoon_age_mos	Age in months when first fed self with a spoon	background_history	16.4 mo (1 – Not yet)	age_fed_self_w_spoon	z-score normalization
**20**	used_words_age_mos	Age in months when first used single words	background_history	16.2 mo (1 – Not yet)	age_used_words	z-score normalization
**21**	combined_words_age_mos	Age in months when first combined words into short phrases or sentences with an action word	background_history	25.3 mo (1 – Not yet)	age_combined_words_short_sen	z-score normalization
**22**	combined_phrases_age_mos	Age in months when first combined phrases into longer sentences	background_history	31.6 mo (1 – Not yet)	NA	z-score normalization
**23**	bladder_trained_age_mos	Age in months when first was bladder-trained (daytime)	background_history	37.3 mo (1 – Not yet)	age_bladder_trained_day	z-score normalization
**24**	bowel_trained_age_mos	Age in months when first was bowel-trained	background_history	39.4 mo (1 – Not yet)	age_bowel_trained	z-score normalization
**25**	hand	Which hand does the child/dependent prefer to use?	background_history	NA	handedness	One-hot encoded
**26**	twin_mult_birth	Is the child/dependent a twin or part of a multiple birth?	background_history	NA	NA	One-hot encoded
**27**	num_asd_parents	Do either of the biological parents have a professional diagnosis of ASD?	background_history	NA	autism_pat or autism_mat	One-hot encoded
**28**	num_asd_siblings	Do you have any full biological siblings who have a professional diagnosis of ASD? If so, how many?	background_history	NA	autism_sibling or autism_y_n	One-hot encoded

eTable 2. Performance of ML Algorithms

The performance of ML algorithms uses a different feature selection and tests the effect of the sex at birth variable as a predictor. The AUC σ value indicates the standard deviation of AUC values across 10-fold cross-validation using the SPARK v8 cohort.

ML Algorithm	Accuracy	AUC	Sensitivity	Specificity	Average Precision	F1-Score
**Features Minimal Medical Screening (N = 30660, ASD = 15330, Non ASD = 15330)**						
**Logistic Regression**	0.707	0.781 (σ = 0.004)	0.615	0.799	0.766	0.677
**Decision Tree**	0.705	0.769 (σ = 0.004)	0.608	0.802	0.745	0.673
**Random Forest**	0.708	0.775 (σ = 0.005)	0.619	0.796	0.755	0.679
**XGBoost**	**0.712**	**0.783 (σ = 0.005)**	**0.628**	**0.796**	**0.767**	**0.686**
**Features Minimal Medical Screening without sex at birth**						
**Logistic Regression**	0.700	0.733	0.559	0.840	0.724	0.650
**Decision Tree**	0.696	0.727	0.550	0.841	0.713	0.644
**Random Forest**	0.698	0.729	0.556	0.839	0.717	0.648
**XGBoost**	**0.700**	**0.735 (σ = 0.005)**	**0.581**	**0.818**	**0.728**	**0.659**
**Features Minimal Background Information**						
**Logistic Regression**	0.698	0.772 (σ = 0.005)	0.726	0.670	0.770	0.706
**Decision Tree**	0.708	0.708 (σ = 0.004)	0.706	0.709	0.647	0.707
**Random Forest**	0.788	0.865 (σ = 0.004)	0.765	0.812	0.867	0.783
**XGBoost**	**0.792**	**0.870 (σ = 0.004)**	**0.763**	**0.821**	**0.874**	**0.786**
**Features Minimal Background Information without sex at birth**						
**Logistic Regression**	0.667	0.726 (σ = 0.005)	0.527	0.808	0.736	0.613
**Decision Tree**	0.696	0.696 (σ = 0.004)	0.697	0.695	0.637	0.697
**Random Forest**	0.781	0.852 (σ = 0.004)	0.747	0.815	0.856	0.773
**XGBoost**	**0.787**	**0.859 (σ = 0.004)**	**0.750**	**0.824**	**0.866**	**0.779**
**Combined model without sex at birth variable**						
**Logistic Regression**	0.735	0.806 (σ = 0.005)	0.661	0.809	0.811	0.714
**Decision Tree**	0.722	0.722 (σ = 0.007)	0.723	0.721	0.660	0.722
**Random Forest**	0.804	0.881(σ = 0.005)	0.792	0.817	0.880	0.802
**XGBoost**	**0.809**	**0.885 (σ = 0.004)**	**0.793**	**0.825**	**0.888**	**0.806**

eTable 3. Performance of the XGBoost Algorithm AutMedAI Stratified by Age

The AUC σ value indicates the standard deviation of AUC values across 10-fold cross-validation using the SPARK v8 cohort.

ML Algorithm (XGBoost)	Accuracy	AUC	Sensitivity	Specificity	Positive Predictive Value	F1-Score
**Features: Medical Screening and Background History**						
Age: 0 - 2 yr (N = 1,088, Autism=544, Non-Autism = 544)	0.789	0.868 (σ = 0.021)	0.771	0.807	0.871	0.785
Age: 2 – 4 yr (N = 3,522, Autism = 1761, Non-Autism = 1761	0.852	0.920 (σ = 0.013)	0.858	0.846	0.913	0.853
Age: 4 – 10 yr (N = 12,820, Autism = 6,410, Non-Autism = 6,410)	0.833	0.906 (σ = 0.004)	0.817	0.849	0.909	0.830
All Ages (N = 30660, Autism = 15330, Non Autism = 15330)	0.817	0.895 (σ = 0.004)	0.805	0.829	0.897	0.815

eTable 4. Performance of the AutMedAI Assessed Using the SPARK Version 10 Cohort (N=11,936, Autism=10,476, Non-Autism=1,460) and stratified by age, race, and sex.

**Experiment**	**Accuracy**	**AUC**	**Sensitivity**	**Specificity**	**Positive Predictive Value**	**F1-Score**
Age: All Ages	0.789	0.790	0.789	0.791	0.964	0.868
All ages without sex at birth	0.765	0.781	0.760	0.803	0.965	0.850
Age: 0 – 2 yr (N=543, Autism=61, Non-Autism=482)	0.785	0.807	0.836	0.778	0.323	0.466
Age: 2 – 4 yr (N=2204, Autism=2031, Non-Autism=173)	0.842	0.798	0.850	0.746	0.975	0.908
Age: 4 - 10 yr (N=5095, Autism=4507, Non-Autism=588)	0.792	0.791	0.793	0.789	0.966	0.871
**Additional experiments**						
Sex: Male	0.826	0.838	0.685	0.967	0.898	
Sex: Female	0.690	0.630	0.891	0.951	0.758	
Race: Asian	0.808	0.812	0.769	0.965	0.882	
Race: African American	0.767	0.769	0.751	0.955	0.852	
Race: Native American	0.743	0.749	0.687	0.959	0.841	
Race: Native Hawaiian	0.843	0.850	0.777	0.973	0.907	
Race: White	0.787	0.786	0.800	0.966	0.866	
Race: Other	0.833	0.831	0.847	0.975	0.897	
Race: Hispanic	0.823	0.833	0.751	0.962	0.893	
Race: More than one	0.788	0.786	0.807	0.966	0.867	

eTable 5. Performance of AutMedAI Using the SSC Cohort With 2854 Individuals With ASD

no non-autistic data available.

Experiment	Accuracy	AUC	Sensitivity	Specificity	Positive Predictive Value	F1-Score
AutMedAI -SSC	0.680	NA	0.680	NA	1.0	NA
AutMedAI -SSC without sex at birth	0.593	NA	0.593	NA	1.0	NA

eTable 6. Differences in Quantitative Measures for Those in the ASD Group (Correct vs Wrong Prediction) and Those in the Non-ASD Group (Correct vs Wrong Prediction)

The results are visualized in Figure 2B.

**Within Autism Prediction group (n=10,476)**	**Record**	**NA**	**Missing**	**Percentage**	**Predicted correctly**	**Predicted wrongly**	**Wilcox test_p-value**
CBCL1-5:total_problems_t_score	1526	57	8893	14,6%	1292	234	2,13E-05
CBCL6-18:total_problems_t_score	2493	8	7975	23,8%	1937	556	7,31E-01
IQ:fsiq_score	547	62	9867	5.2%	415	132	5,04E-08
SCQ:final_score	9350	200	926	89,3%	7350	2000	1,77E-159
**Within the non-autism Prediction group (n=1,460)**	**Record**	**NA**	**Missing**	**Percentage**	**Predicted correctly**	**Predicted wrongly**	**Wilcox test_p-value**
CBCL1-5:total_problems_t_score	0	0	1460	0%	NA	NA	NA
CBCL6-18:total_problems_t_score	0	0	1460	0%	NA	NA	NA
IQ:fsiq_score	0	0	1460	0%	NA	NA	NA
SCQ:final_score	1280	40	140	87,7%	1006	274	3,52E-19

eTable 7. Frequency and Odds Ratio for Different Behavioral and Developmental Diagnoses Between Those Those in the ASD Group (Correct vs Wrong Prediction) and Those in the Non-ASD Group (Correct vs Wrong Prediction)

Outcome	Prediction Result	Phenotype	Type	Num with	Num without	Num all	Feq	Chisq_p(Freq)	Chisq_p_adjust(=Chisq_p*11)	significance(Freq p)	OR(Yes:No)	lower_95 %CI	upper_95 %CI
**Autism**	Yes	Attention or behavior disorders	Behave	3431	4831	8262	0,415	9,76E-16	1,073E-14	***	0,68	0,62	0,75
**Autism**	Yes	Attention Deficit-Hyperactivity Disorder	Behave	2821	5441	8262	0,341	2,64E-25	2,905E-24	***	0,60	0,55	0,67
**Autism**	Yes	Conduct Disorder	Behave	175	8087	8262	0,021	3,80E-02	4,183E-01	NA	1,52	1,05	2,27
**Autism**	Yes	Intermittent Explosive Disorder	Behave	174	8088	8262	0,021	2,89E-02	3,182E-01	NA	1,56	1,07	2,35
**Autism**	Yes	Oppositional Defiant Disorder	Behave	551	7711	8262	0,067	1,55E-04	1,702E-03	**	0,72	0,61	0,85
**Autism**	Yes	Language delay or language disorder	Develop ment	5096	3166	8262	0,617	8,58E-184	9,437E-183	***	4,31	3,89	4,78
**Autism**	Yes	Learning disability	Develop ment	2025	6237	8262	0,245	3,44E-24	3,781E-23	***	1,93	1,70	2,20
**Autism**	Yes	Motor delay or developmental coordination disorder	Develop ment	1648	6614	8262	0,199	5,86E-57	6,442E-56	***	4,16	3,46	5,04
**Autism**	Yes	Mutism	Develop ment	113	8149	8262	0,014	2,68E-01	2,945E+00	NA	1,31	0,85	2,11
**Autism**	Yes	Social (Pragmatic) Communication Disorder	Develop ment	1538	6724	8262	0,186	8,33E-23	9,161E-22	***	2,10	1,81	2,45
**Autism**	Yes	Speech articulation problems	Develop ment	2457	5805	8262	0,297	9,48E-27	1,043E-25	***	1,89	1,68	2,13
**Non-Autism**	Yes	Attention or behavior disorders	Behave	200	955	1155	0,173	7,41E-02	8,156E-01	NA	0,74	0,55	1,02
**Non-Autism**	Yes	Attention Deficit-Hyperactivity Disorder	Behave	183	972	1155	0,158	1,31E-01	1,442E+00	NA	0,77	0,56	1,07
**Non-Autism**	Yes	Conduct Disorder	Behave	4	1151	1155	0,003	1,11E-01	1,218E+00	NA	0,26	0,06	1,17
**Non-Autism**	Yes	Intermittent Explosive Disorder	Behave	6	1149	1155	0,005	1,00E+00	1,100E+01	NA	0,75	0,17	5,72
**Non-Autism**	Yes	Oppositional Defiant Disorder	Behave	26	1129	1155	0,023	4,11E-01	4,524E+00	NA	0,67	0,33	1,49
**Non-Autism**	Yes	Language delay or language disorder	Develop ment	52	1103	1155	0,045	1,36E-26	1,497E-25	***	0,15	0,10	0,22
**Non-Autism**	Yes	Learning disability	Develop ment	30	1125	1155	0,026	1,35E-03	1,484E-02	*	0,38	0,21	0,69
**Non-Autism**	Yes	Motor delay or developmental coordination disorder	Develop ment	14	1141	1155	0,012	7,59E-06	8,349E-05	***	0,21	0,10	0,43
**Non-Autism**	Yes	Mutism	Develop ment	1	1154	1155	0,001	1,00E+00	1,100E+01	NA			
**Non-Autism**	Yes	Social (Pragmatic) Communication Disorder	Develop ment	3	1152	1155	0,003	5,75E-04	6,328E-03	**			

**Non-Autism**	Yes	Speech articulation problems	Develop ment	51	1104	1155	0,044	1,34E-07	1,478E-06	***			
**Autism**	No	Attention or behavior disorders	Behave	1131	1083	2214	0,511	9,76E-16	1,073E-14	***	0,68	0,62	0,75
**Autism**	No	Attention Deficit-Hyperactivity Disorder	Behave	1022	1192	2214	0,462	2,64E-25	2,905E-24	***	0,60	0,55	0,67
**Autism**	No	Conduct Disorder	Behave	31	2183	2214	0,014	3,80E-02	4,183E-01	NA	1,52	1,05	2,27
**Autism**	No	Intermittent Explosive Disorder	Behave	30	2184	2214	0,014	2,89E-02	3,182E-01	NA	1,56	1,07	2,35
**Autism**	No	Oppositional Defiant Disorder	Behave	200	2014	2214	0,090	1,55E-04	1,702E-03	**	0,72	0,61	0,85
**Autism**	No	Language delay or language disorder	Develop ment	602	1612	2214	0,272	8,58E-184	9,437E-183	***	4,31	3,89	4,78
**Autism**	No	Learning disability	Develop ment	318	1896	2214	0,144	3,44E-24	3,781E-23	***	1,93	1,70	2,20
**Autism**	No	Motor delay or developmental coordination disorder	Develop ment	125	2089	2214	0,056	5,86E-57	6,442E-56	***	4,16	3,46	5,04
**Autism**	No	Mutism	Develop ment	23	2191	2214	0,010	2,68E-01	2,945E+00	NA	1,31	0,85	2,11
**Autism**	No	Social (Pragmatic) Communication Disorder	Develop ment	217	1997	2214	0,098	8,33E-23	9,161E-22	***	2,10	1,81	2,45
**Autism**	No	Speech articulation problems	Develop ment	405	1809	2214	0,183	9,48E-27	1,043E-25	***	1,89	1,68	2,13
**Non-Autism**	No	Attention or behavior disorders	Behave	67	238	305	0,220	7,41E-02	8,156E-01	NA	0,74	0,55	1,02
**Non-Autism**	No	Attention Deficit-Hyperactivity Disorder	Behave	60	245	305	0,197	1,31E-01	1,442E+00	NA	0,77	0,56	1,07
**Non-Autism**	No	Conduct Disorder	Behave	4	301	305	0,013	1,11E-01	1,218E+00	NA	0,26	0,06	1,17
**Non-Autism**	No	Intermittent Explosive Disorder	Behave	2	303	305	0,007	1,00E+00	1,100E+01	NA	0,75	0,17	5,72
**Non-Autism**	No	Oppositional Defiant Disorder	Behave	10	295	305	0,033	4,11E-01	4,524E+00	NA	0,67	0,33	1,49
**Non-Autism**	No	Language delay or language disorder	Develop ment	73	232	305	0,239	1,36E-26	1,497E-25	***	0,15	0,10	0,22
**Non-Autism**	No	Learning disability	Develop ment	20	285	305	0,066	1,35E-03	1,484E-02	*	0,38	0,21	0,69
**Non-Autism**	No	Motor delay or developmental coordination disorder	Develop ment	17	288	305	0,056	7,59E-06	8,349E-05	***	0,21	0,10	0,43
**Non-Autism**	No	Mutism	Develop ment	0	305	305	0,000	1,00E+00	1,100E+01	NA			
**Non-Autism**	No	Social (Pragmatic) Communication Disorder	Develop ment	7	298	305	0,023	5,75E-04	6,328E-03	**			
**Non-Autism**	No	Speech articulation problems	Develop ment	39	266	305	0,128	1,34E-07	1,478E-06	***			

eFigure 1. Overview of the Study and the ML Model Development

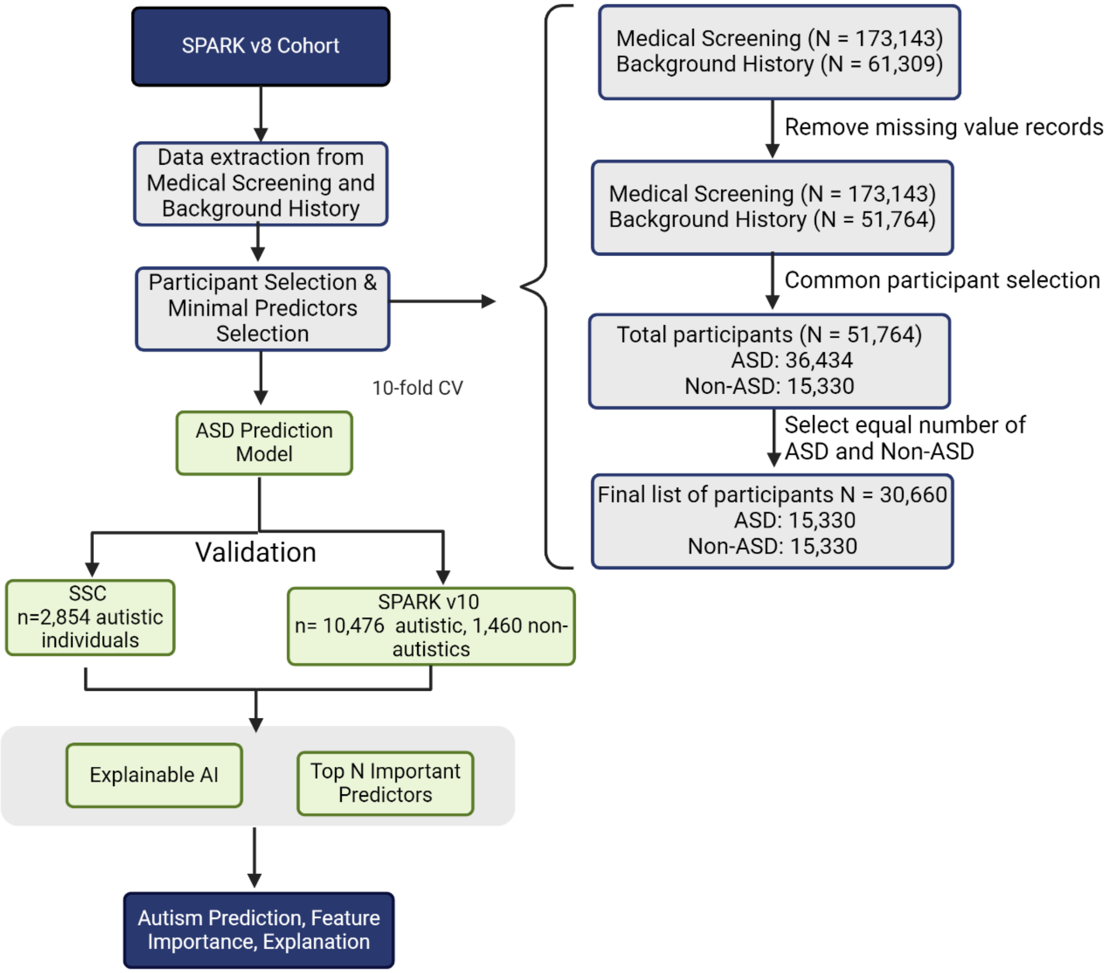

A flow diagram to illustrate the pipeline and approach, including the participant selection process prior to model training.

eFigure 2. Influencing Predictors for Autism Detection in the Group Aged 0 to 2 Years

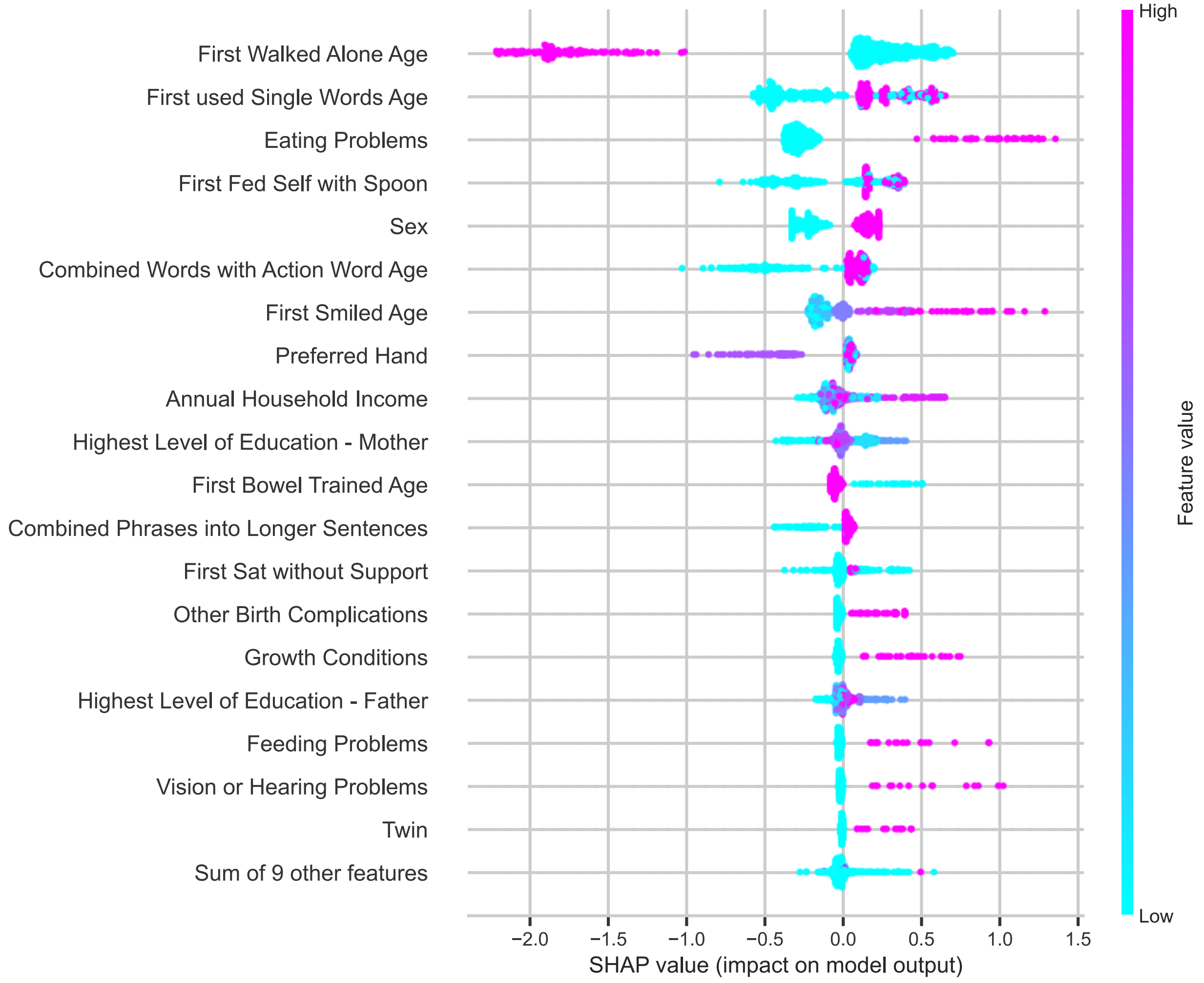

A beeswarm plot for all test set samples in the age group of 0 – 2 years of the SPARK v10 cohort shows SHAP values for features. The x-axis represents the features' contribution to the final model prediction. A positive or negative value is associated with an increase in the likelihood of predicting autism or non-autism, respectively. Every point in the plot represents a test sample. The color gradient indicates the range of feature values.

eFigure 3. A Beeswarm Plot for All Test Set Samples in the SPARK Version 10 Cohort Aged 2 to 4 Years

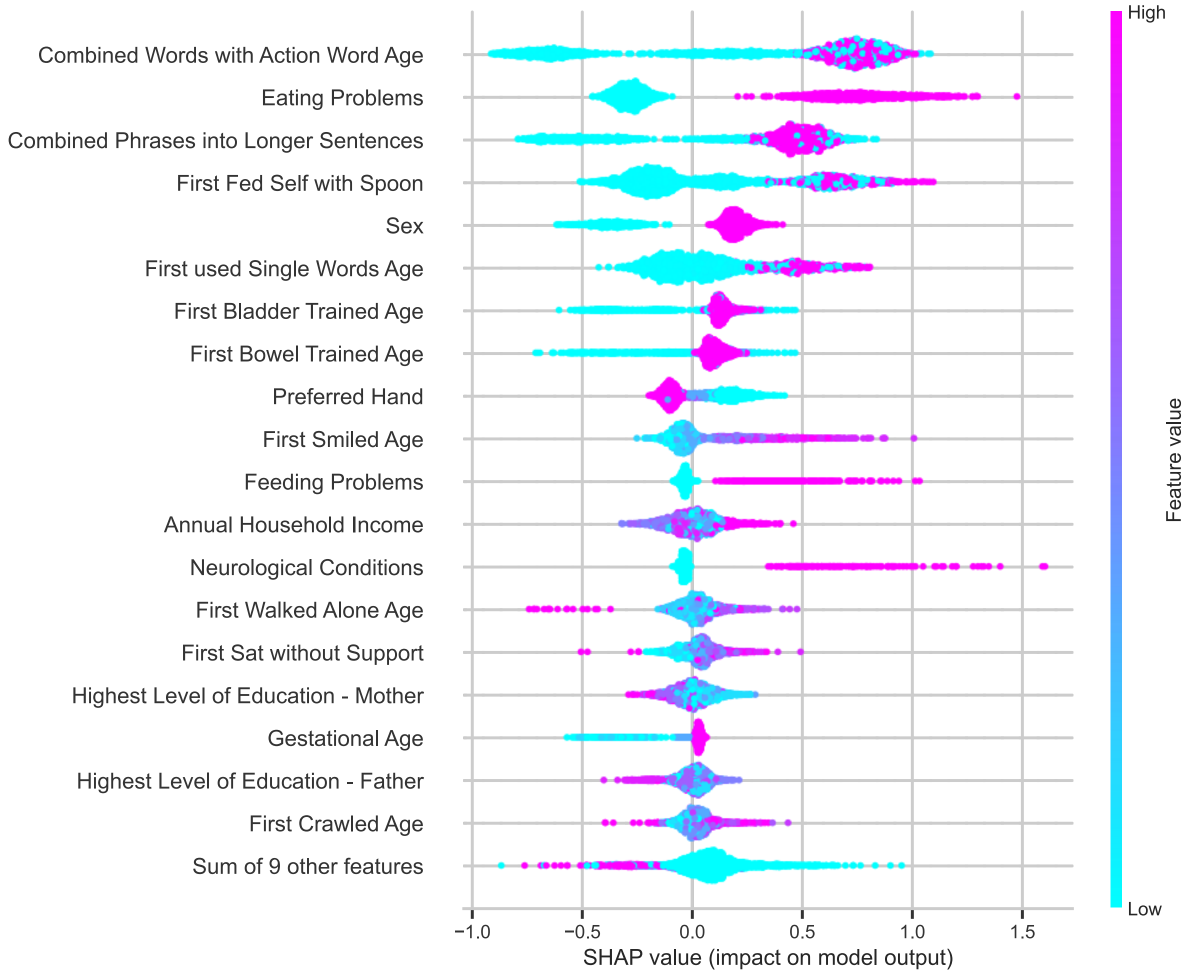

A beeswarm plot for all test set samples in the 2 – 4 years of SPARK v10 cohort shows SHAP values for features. The x-axis represents the features' contribution to the final model prediction. The positive or negative value is associated with an increase in the likelihood of predicting autism or non-autism, respectively. Every point in the plot represents a test sample. The color gradient indicates the range of feature values.

eFigure 4. A Beeswarm Plot for all Test Set Samples in the SPARK Version 10 Cohort Aged 4 to 10 Years

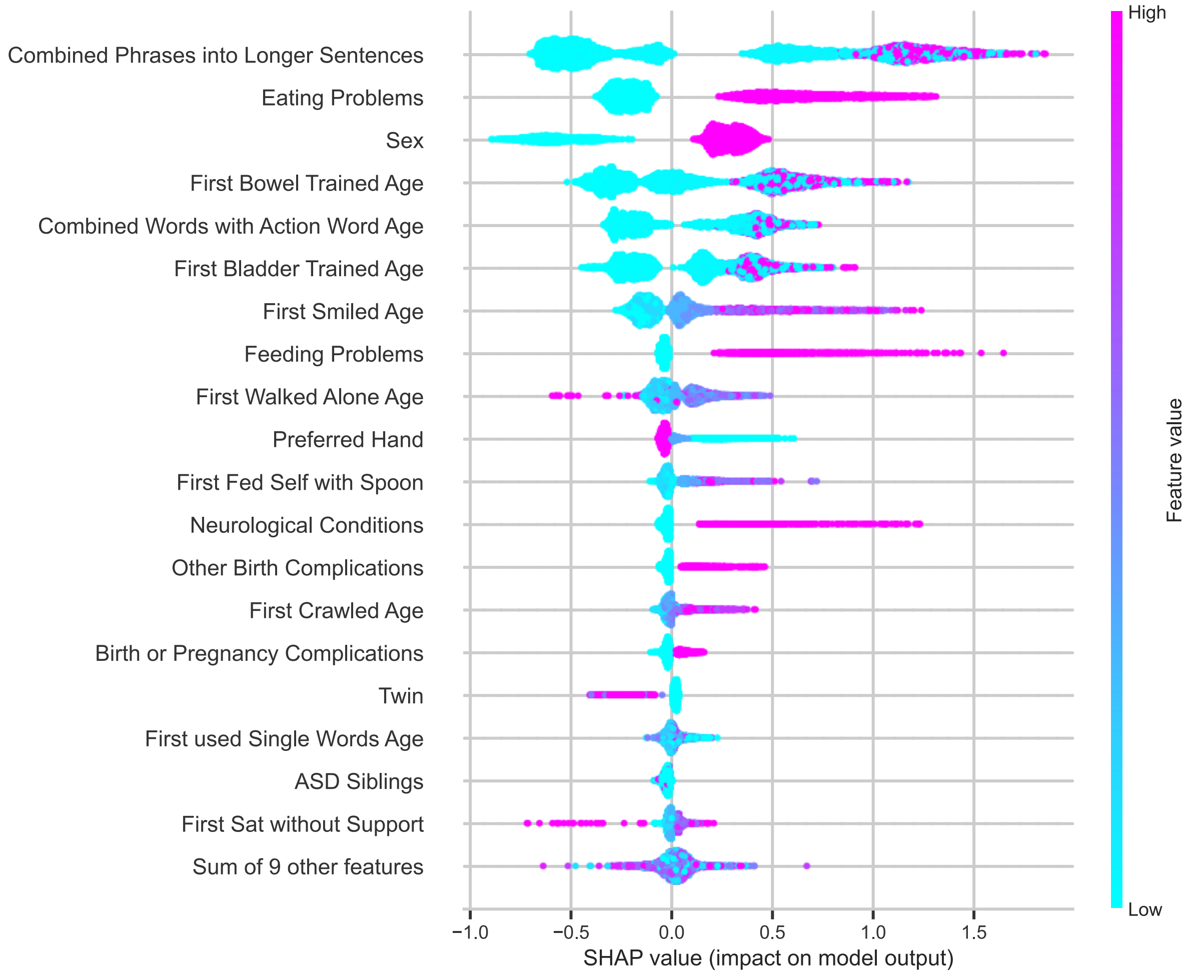

A Beeswarm plot for all test set samples in the age group of 4 – 10 years of the SPARK v10 cohort showing SHAP values for features. The x-axis represents features' contribution to the final model prediction. A positive or negative value is associated with an increase in the likelihood of predicting autism or non-autism, respectively. Every point in the plot represents a test sample. The color gradient indicates the range of feature values.

eFigure 5. A Beeswarm Plot for Test Set Samples of the SPARK Version 10 Cohort

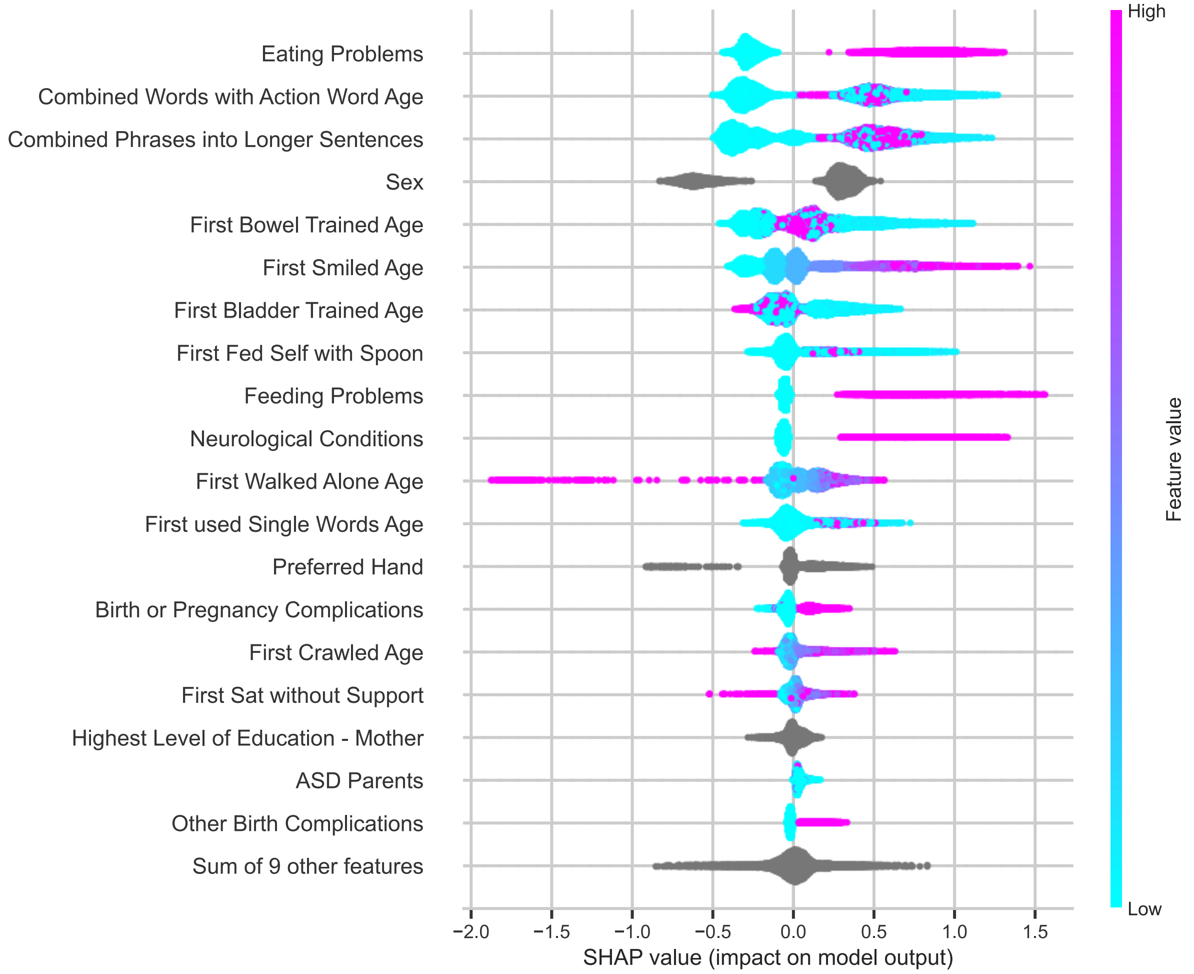

A Beeswarm plot for test set samples of the SPARK v10 cohort showing Shapley additive explanations (SHAP) values for features. The x-axis represents the features' contribution to the final model prediction. The positive or negative value is associated with an increase in the likelihood of predicting autism or non-autism, respectively. Every point in the plot represents a single participant. The color gradient indicates the range of feature values.

eFigure 6. The Influence of the Top 20 Features for 6 Individuals in the SPARK Version 10 Cohort

A) autism – prediction of autism

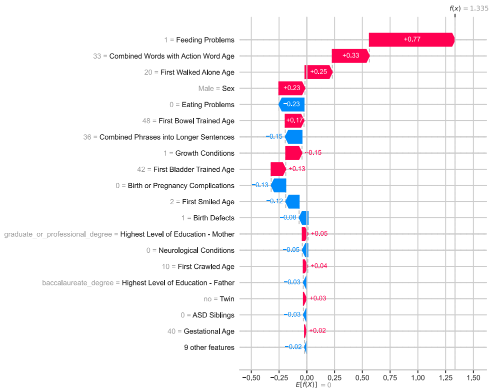

B) autism – prediction of autism

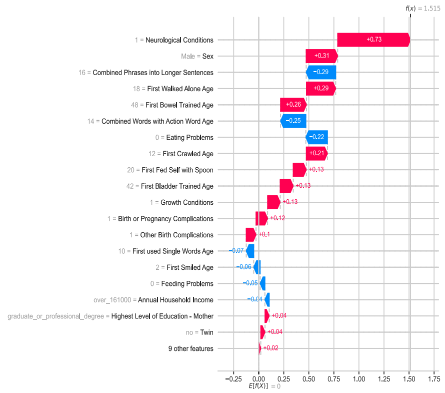

C) autism – prediction of autism

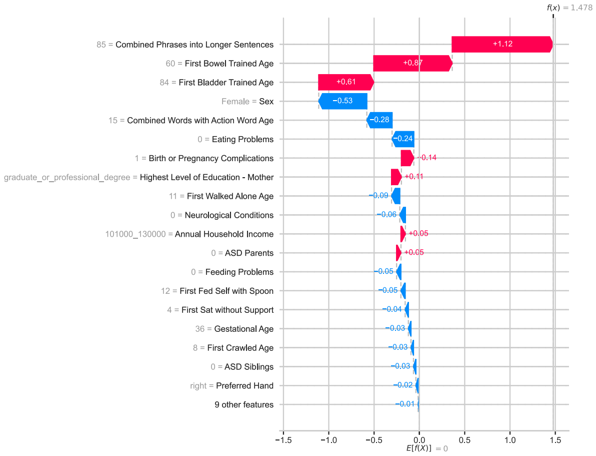

D) autism – prediction non-autism

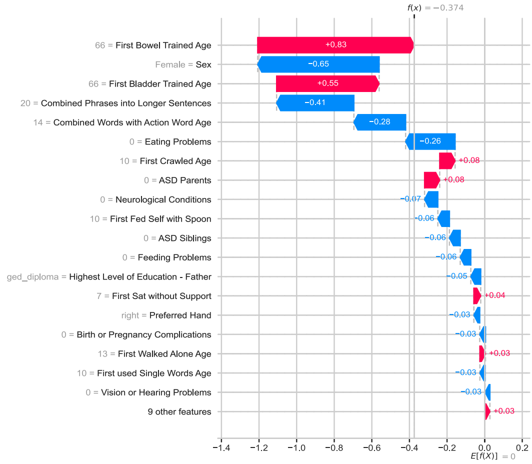

E) autism – prediction non-autism

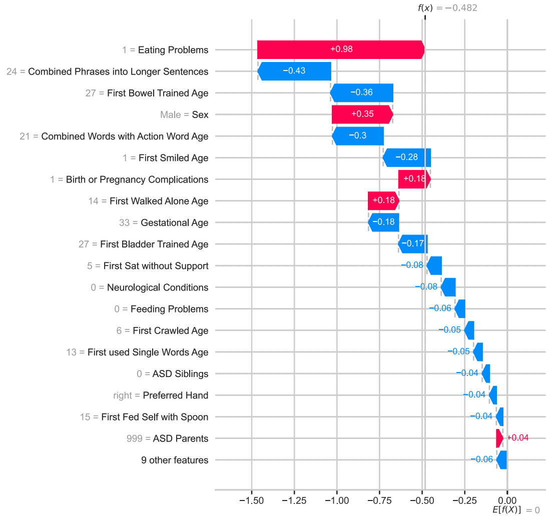

F) autism – prediction non-autism

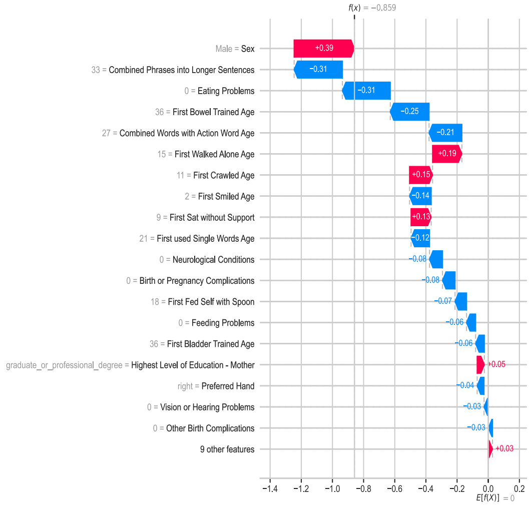

. (A – C) Figures represent these features and their influence on three individuals diagnosed with autism, and the proposed XGBoost “AutMedAI” ML model prediction was correct. (D – F) Figures represent these features and their influence in three individuals diagnosed with autism, and the proposed XGBoost “AutMedAI” ML model was incorrectly predicted as non-Autism. The x-axis represents SHAP values, and the y-axis lists the top 20 features. The red colored bar against each feature indicates prediction towards Autism, with SHAP values written inside the bar indicating the extent of influence towards Autism prediction. The blue bar with negative values indicates the features influencing the reverse direction, i.e., towards non-autism prediction. The grey values before the feature names are the actual feature values for an individual.

eFigure 7. Decision Plot Showing the Influence of Features on Model Prediction

A)

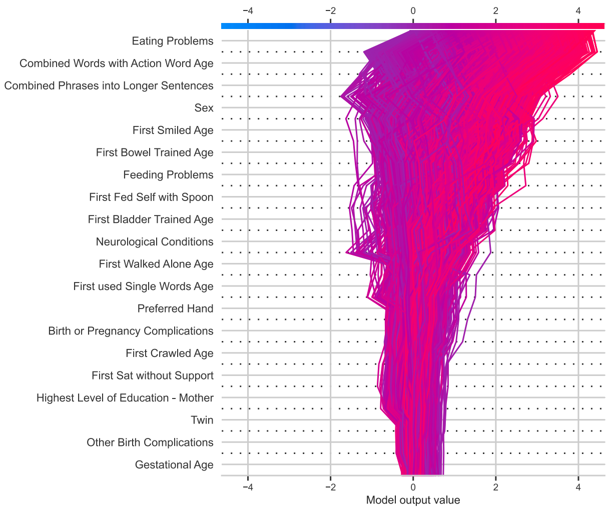

B)

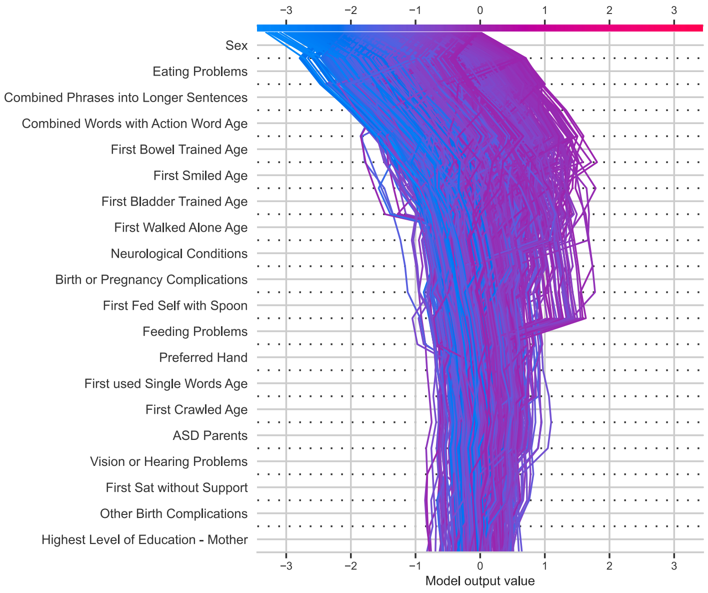

A) All correctly predicted autistic individuals. (b) All incorrectly predicted autistic individuals. Each line indicates the contribution of all features toward the model output. The X-axis indicates model output. The Y-axis lists all features from the top in decreasing order of importance. A colored line represents each sample. The curve is inclined towards a positive output value, indicating an autism prediction. The curve is inclining towards a negative value, thereby incorrectly predicting non-autism.
