# Supplementary material for: Machine Learning Prediction of Autism Spectrum Disorder From a Minimal Set of Medical and Background Information
Source: JAMA Netw Open. 2024 Aug 19;7(8):e2429229. doi: 10.1001/jamanetworkopen.2024.29229 (PMC11333987; doi:10.1001/jamanetworkopen.2024.29229)
Supplement: Supplement 2. — Data Sharing Statement [file jamanetwopen-e2429229-s002.pdf]

Data Sharing Statement

Rajagopalan. Machine Learning Prediction of Autism Spectrum Disorder From a Minimal Set of Medical and Background Information. *JAMA Netw Open*. Published August 19, 2024. doi:10.1001/jamanetworkopen.2024.29229

Data
Data available: Yes
Data types: Deidentified participant data
How to access data: The whole dataset used in the study is available through the Simons Foundation database for autism research (SFARI base https://www.sfari.org/resource/sfari-base). Details about the cohorts used in this study are in the manuscript.
When available: With publication

Supporting Documents
Document types: Statistical/analytic code
How to access documents: the information is available in the manuscript.
When available: With publication

Additional Information
Who can access the data: The code will be available for everyone. The procedure for getting access to the data is available in the website of SFARI base.
Types of analyses: The code will be available for everyone. The procedure for getting access to the data is available in the website.
Mechanisms of data availability: The code will be available for everyone. The procedure for getting access to the data is available in the website.
Any additional restrictions: The code will be available for everyone. The procedure for getting access to the data is available in the website.
